# Prediction of benefit from docetaxel chemotherapy for HR-positive breast cancer in the PACS-01 trial

**DOI:** 10.1016/j.breast.2026.104894

**Published:** 2026-08-07

**Authors:** Frédérique Penault-Llorca, Amelie Lusque, Thomas Filleron, Kevin Tran, Lili Du, Rick Baehner, Florence Dalenc, Magali Lacroix-Triki, Thomas Bachelot, Fabrice Andre, Pascal Boucher, Jérôme Lemonnier, W. Fraser Symmans

**Affiliations:** a Department of Biopathology, Centre Jean Perrin, Université Clermont Auvergne, INSERM, U1240 Imagerie Moléculaire et Stratégies Théranostiques, Clermont Ferrand, F-63000, France; b Biostatistics & Health Data Science Unit, University of Toulouse, Oncopole Claudius Regaud, IUCT-Oncopole, Toulouse, France; c Department of Translational Molecular Pathology, University of Texas MD Anderson Cancer Center, Houston, TX, USA; d Exact Sciences, Madison, WI, USA; e Department of Pathology, University of California, San Francisco, CA, USA; f Department of Medical Oncology, Oncopole Claudius Regaud, Institut Universitaire du Cancer Toulouse-Oncopole, Toulouse, France; g Department of Pathology, Institut Gustave Roussy, Villejuif, France; h Department of Medical Oncology, Centre Leon Berard, Lyon, France; i Department of Medical Oncology, Institut Gustave Roussy, Villejuif, France; j Unicancer, Paris, France

**Keywords:** Docetaxel, Anthracycline, Chemotherapy, Prediction, Hormone receptor, SET, Index, Recurrence score, Menopausal, Breast cancer, Endocrine, Transcription, Activity

## Abstract

**Background:**

Adjuvant chemotherapy for hormone receptor–positive (HR+) breast cancer relies on taxanes, but existing tests do not identify which patients benefit from them. The SET_ER/PR_ index, a measure of endocrine-related transcriptional activity in HR + tumors, recently predicted benefit from paclitaxel when low (<0.75), but not for anthracycline-based therapy. We tested whether SET_ER/PR_ could predict benefit from docetaxel in node-positive, HR + patients enrolled in the PACS-01 trial (docetaxel after fluorouracil–epirubicin–cyclophosphamide (3FEC+3D) versus 6FEC).

**Patients and methods:**

SET_ER/PR_ index and Recurrence Score (RS) were obtained for 490 patients. SET_ER/PR_ was quantified using the QuantiGene Plex assay from RNA remaining after RS testing. Pre-specified analyses assessed SET_ER/PR_ as a continuous variable and using the predefined <0.75 cut point, as well as continuous RS and the >25 cut point. The primary endpoint was distant recurrence-free interval (DRFI). Predictive value was assessed using Cox models including biomarker, treatment arm, and interaction term.

**Results:**

Continuous SET_ER/PR_ demonstrated significant interaction with treatment on DRFI (P_interaction_ = 0.028), whereas predefined <0.75 cut point was not predictive (P_interaction_ = 0.668). Exploratory analyses identified a cut point at 1.50 (P_interaction_ = 0.013): patients with SET_ER/PR_ ≥ 1.50 had worse outcomes with 3FEC+3D versus 6FEC (HR 3.16, 95%CI 1.28–7.85), while outcomes were similar between arms when SET_ER/PR_ < 1.50 (HR 0.83, 95%CI 0.51–1.35). RS did not predict differential benefit, either continuously (P_interaction_ = 0.670) or at the >25 threshold (P_interaction_ = 0.534).

**Conclusion:**

Including sequential docetaxel (3FEC+3D) was less effective than continued anthracycline chemotherapy (6FEC) when breast cancer had high endocrine-related transcriptional activity (i.e., SET_ER/PR_ index ≥1.50).

**Trial registration:**

PACS-01.

## Highlights

• Predictive interaction between SET_ER/PR_ index of endocrine transcriptional activity and efficacy of docetaxel.
• Wherein addition of docetaxel was inferior to continuing the anthracycline treatments for patients with high SET_ER/PR_ index.
• Current standards, i.e., Recurrence Score, nodal burden, and menopausal status did not demonstrate any predictive interaction.

## Graphical abstract

Graphical abstracts should be submitted as a separate supplementary file in the online submission system. Image size: Please provide an image with a minimum of 531 × 1328 pixels (h X w) or proportionally more. The image should be readable at a size of 5 × 13 cm using a regular screen resolution of 96 dpi. Preferred file types: TIFF, EPS, PDF or MS Office files.

**Figure:**
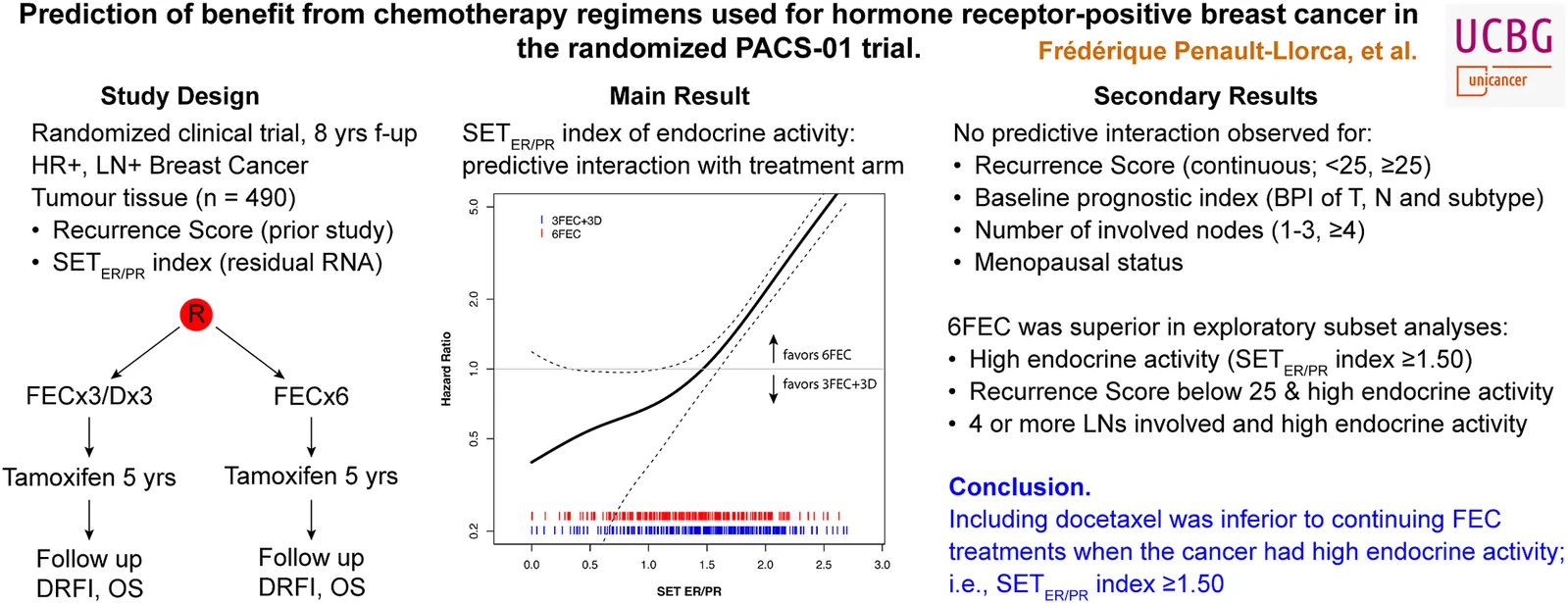

## 1 Introduction

Information about the burden of disease, genomic test results and menopausal status are considered when selecting which patients with hormone receptor-positive (HR-positive) cancer should be encouraged to receive adjuvant chemotherapy [[1], [2], [3]]. A recent meta-analysis of randomized trials conducted by the Early Breast Cancer Trials Collaborative Group (EBCTCG) demonstrated that the inclusion of taxanes in adjuvant chemotherapy achieved similar relative benefit in HR-positive and HR-negative breast cancer, with no difference by menopausal status [3]. Of course, the EBCTCG meta-analysis did not have data relating to contemporary genomic predictive tests. It is possible that the additional benefit from including a taxane is generalized, i.e., that chemotherapy benefit in all patients is augmented by this improved regimen. However, it is also possible that chemosensitivity depends more specifically on the type of regimen administered and the biological characteristics of a HR-positive breast cancer.

A general chemo-predictive biomarker would inform whether, or not chemotherapy is generally likely to benefit a patient, whereas a specific chemopredictive biomarker would inform which chemotherapy regimen would offer more, or less benefit. The Recurrence Score (RS) is the most qualified example of a general chemo-predictive test because it was validated in randomized comparisons of adjuvant chemotherapy versus no chemotherapy [4,5]. In particular, two phase 3 prospective trials used RS results to identify patients who were randomized patients to receive adjuvant chemotherapy (CT), or not, prior to adjuvant endocrine therapy (ET): TAILORx demonstrated that ET alone was not inferior to CT + ET for node-negative cancer with RS in the range 11–25; and RxPONDER demonstrated no significant benefit from CT + ET compared to ET alone for postmenopausal patients whose cancer had 1–3 lymph nodes involved and RS ≤ 25, but there was benefit from CT for premenopausal patients [1,2]. Interestingly, RS and other biomarkers that reported general chemo-predictive performance for HR-positive breast cancer have not yet predicted when a specific chemotherapy regimen offers greater or less benefit [[6], [7], [8], [9], [10]].

To our knowledge, the sensitivity to endocrine therapy (SET_ER/PR_) index is the first genomic assay to predict outcomes between specific chemotherapy regimens for HR-positive breast cancer. SET_ER/PR_ index measures endocrine activity in the cancer from gene transcription related to estrogen (ER) and progesterone receptors (PR), but not proliferation [11,12]. This assay yields reproducible results within and between different laboratories [13,14]. A report from the CALGB 9741 trial demonstrated that low SET_ER/PR_ index (below 0.75) in HR-positive cancers predicted survival benefit from dose-dense (2-weekly), versus conventional 3-weekly dosing schedule of the same anthracycline and paclitaxel treatments [15]. A second study of HR-positive/HER2-negative cancers from the GEICAM/9906 trial demonstrated that low SET_ER/PR_ index (below 0.75) predicted survival benefit from including weekly paclitaxel after conventional dosing of anthracycline treatments, versus continued treatments with the anthracycline regimen [16]. However, SET_ER/PR_ index was not associated with benefit from conventional dosing of anthracycline treatments, versus no chemotherapy in the SWOG 8814 trial, suggesting that prediction in CALGB 9741 might be related to paclitaxel, or dose-density in general [[15], [16], [17]].

In this study we evaluated the SET_ER/PR_ index for prediction of benefit from docetaxel in patients with, HR + cancer from the randomized PACS-01 trial. PACS-01 was a pivotal phase-3 trial that established clinical utility for sequential combination of three cycles of fluorouracil, epirubicin and cyclophosphamide followed by 3 cycles of docetaxel (3FEC+3D), versus six cycles of FEC (6FEC) as adjuvant chemotherapy for node-positive breast cancer [18,19].

## 2 Methods

### 2.1 Objectives

Our hypothesis was that addition of docetaxel would be more effective in cancers with lower level of endocrine activity (using SET_ER/PR_ index) than in cancers with higher endocrine activity. The SET_ER/PR_ index had not been studied previously for prediction of a benefit from docetaxel, so there was no cut point known and the prespecified approach was to evaluate the continuous SET_ER/PR_ index for predictive interaction with treatment arm (Supplementary Appendix, section 4.5.2). A second objective was to evaluate predictive associations with RS (≤25 versus >25, and as a continuous score in 10-point increments), a baseline prognostic index that combines pathologic findings of tumor size, number of involved lymph nodes, and molecular subtype score [15,20]; and relevant clinical characteristics (Supplementary Appendix, section 4.5.4).

### 2.2 Patients

The UNICANCER PACS-01 trial was a multicenter, open label, randomized phase III trial comparing two adjuvant chemotherapy regimens after breast cancer surgery in 1999 women with lymph node–positive disease [18]. The patients were treated either with six 21-day cycles of 5-fluorouracil, epirubicin and cyclophosphamide (6FEC; control arm) or with three 21-day cycles of FEC followed by three 21-day cycles of docetaxel (3FEC+3D; experimental arm) [18]. Adjuvant endocrine therapy was per standard of care, which was initially only approved for post-menopausal women but became standard for premenopausal women while PACS-01 was accruing patients [18]. The median follow-up duration for the whole cohort was 8 years ^17.^ Among the 1999 patients included in the study, 1366 had HR-positive breast cancer and received adjuvant ET (tamoxifen) [19]. [19]. Details of the study design and the patients’ characteristics were published previously [20].

The PACS-01 trial was performed in accordance with the Declaration of Helsinki and approved by the ethics committees at all participating institutions, patients provided their written informed consent for therapy randomization and future research using their tumor samples. Formalin-fixed, paraffin-embedded (FFPE) blocks from each patient's primary tumor were prepared at the time of surgery. Total RNA had been prepared from these tumor samples for previous study [10], and the residual RNA was used to measure the SET_ER/PR_ index and BPI using the Quantigene Plex hybridization assay, using previously reported methods [[13], [14], [15],^20^].

### 2.3 Materials

The current study follows a formal prospective–retrospective design per REMARK criteria and per the guidelines for use of archived clinical trial specimens for predictive biomarker evaluation on clinical trials [21,22]. The analysis population was defined as patients with HR + breast cancer who had both SET_ER/PR_ index and RS genomic results and who had received adjuvant endocrine therapy, as defined in our recent publication on prognostic outcomes [20]. Briefly, tumor blocks had been sent to Genomic Health (now Exact Sciences, Madison, WI) to perform the Recurrence Score (RS) gene expression assay [10], and in 2022 the residual RNA was sent to the MDACC laboratory to measure SET_ER/PR_ index under an approved material transfer agreement and with ethics approval [20]. The investigators remained blinded to sample identity and subject's treatment arm and outcome, except for the UNICANCER statisticians who prepared the analysis plan and independently conducted the analyses.

### 2.4 Statistical

The primary endpoint of distant recurrence-free interval (DRFI) was decided in advance and is defined as the time from the date of random assignment to the first date of diagnosis of distant recurrence [23]. The secondary endpoint was overall survival (OS), defined as the time from randomization to the date of death from any cause. Subset analyses addressed the clinically-relevant chemoprediction categories of high and low RS that were used in the RxPONDER trial (i.e., RS > 25 and RS ≤ 25); menopausal status, because results from RxPONDER trial differed by menopausal status); and nodal stage, because there are currently insufficient published results about predictive biomarkers for patients with ≥4 positive lymph nodes [2,24,25].

Data were summarized by median and range (min-max) or interquartile range (Q1-Q3) for continuous variables and by frequencies and percentages for categorical variables. Comparisons between groups were performed using Chi-squared and Mann-Whitney tests for categorical and continuous variables, respectively. The interaction between biomarker and treatment arm (3FEC+3D versus 6FEC) was assessed using a Cox proportional hazards model including biomarker, treatment arm and their interaction term for DRFI and OS. Kaplan-Meier curves were used to estimate and compare the survival distribution across groups. The Log-rank test was used to assess the association between treatment arm and survival endpoint. The hazard ratios (HR) were computed with 95% confidence interval (95%CI) using Cox proportional hazards models. Subgroup analyses were summarized in Forest plots. All statistical tests were two-sided and the level of significance was set at 0.05. Statistical analyses were conducted using Stata v18 and R v4.1.2 software.

## 3 Results

### 3.1 Analysis population

**Fig. 1:**
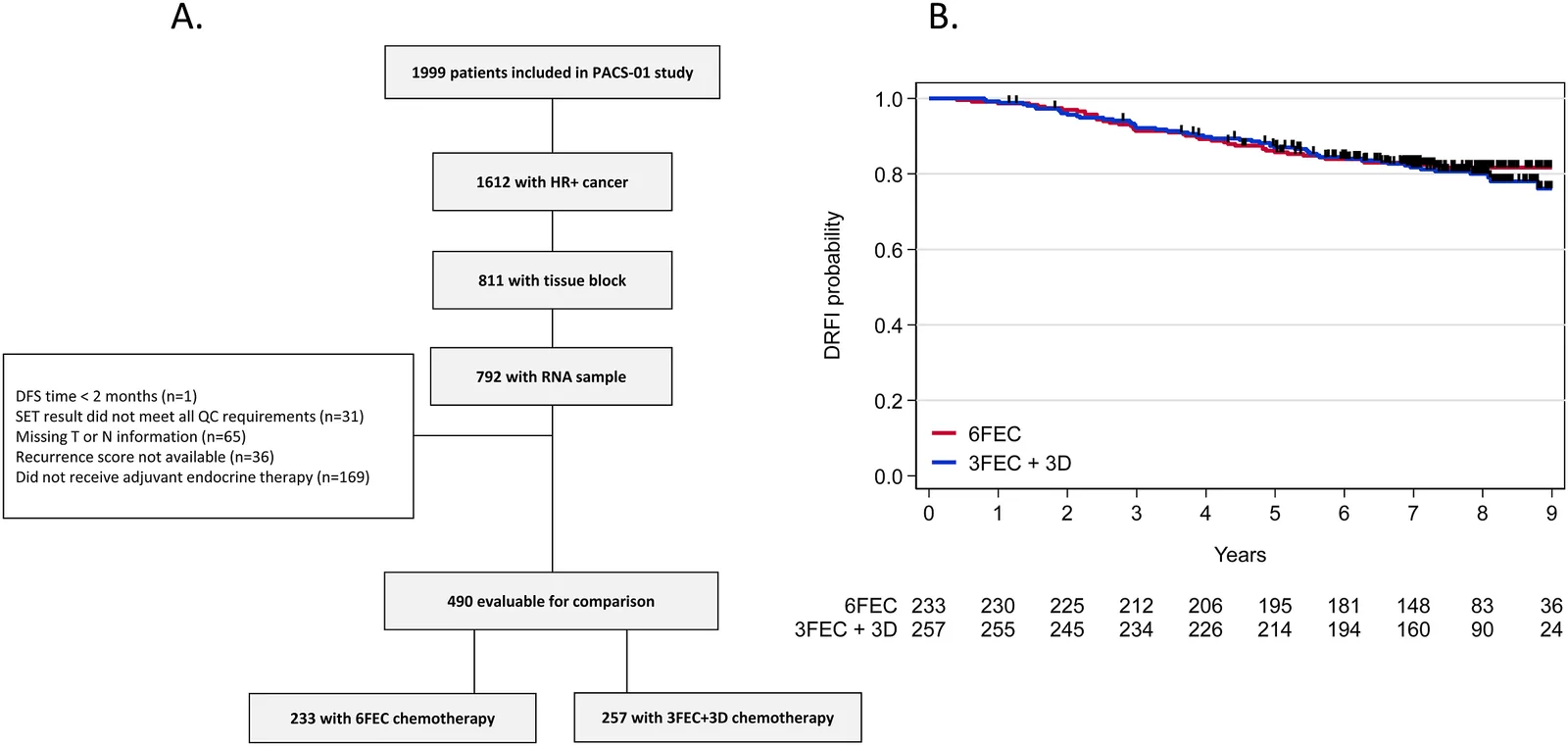
A, REMARK diagram of samples from PACS-01 trial patients evaluated in this translational study. B, Kaplan-Meier plot of distant recurrence-free interval (DRFI) for the 490 patients evaluable in this study, according to treatment arm. Abbreviations: HR, hormone receptor; SET, sensitivity to endocrine therapy biomarker; QC, quality control; T, tumor size; N, number of involved lymph nodes; 6FEC, six cycles of 5-fluorouracil, epirubicin, and cyclophosphamide; 3FEC+3D, three cycles of 5-fluorouracil, epirubicin, and cyclophosphamide followed by three cycles of docetaxel.

Patients' characteristics were well balanced between treatment arms in this study (Table 1). Fig. 1A shows a REMARK diagram of participants and samples used in this study, leading to the 490 that were eligible for this planned comparison of adjuvant chemotherapy regimens in patients who also received adjuvant endocrine therapy and had the required information from all genomic indices (SET_ER/PR_, BPI and RS), the same population that was reported in our recent publication assessing prognostic performance [20]. At a median follow-up of 8 years, 94/490 patients (19%) experienced distant recurrence, and death was recorded during follow-up in 70 of 490 patients (14%). Patients’ outcomes between treatment arms (3FEC+3D versus 6FEC) did not differ for DRFI (HR 1.13, 95%CI 0.75-1.70), with Kaplan-Meier plot shown in Fig. 1B, or for overall survival (HR 0.90, 95%CI 0.57-1.45).

**Table 1:** Patient and baseline characteristics in the study cohort and by treatment arm.

	Total	6FEC	3FEC+3D	p-value
	(N = 490)	(N = 233)	(N = 257)	
**Age (Years)**				
Median (Range)	51 (30; 64)	51 (30; 64)	51 (31; 64)	0.348
<50 Years	196 (40%)	98 (42%)	98 (38%)	0.375
≥50 years	294 (60%)	135 (58%)	159 (62%)	
**Menopausal Status**				
Premenopausal	258 (53%)	122 (53%)	136 (53%)	0.969
Postmenopausal	225 (47%)	106 (47%)	119 (47%)	
Missing	7	5	2	
**Surgery**				
Breast conservation	303 (62%)	143 (61%)	160 (62%)	0.841
Mastectomy	187 (38%)	90 (39%)	97 (38%)	
**Histologic Tumor Size (mm)**				
Median (Range)	20 (3; 150)	20 (5; 150)	20 (3; 100)	0.778
≤20 mm	256 (52%)	127 (55%)	129 (50%)	0.340
>20 mm	234 (48%)	106 (45%)	128 (50%)	
**Number of involved Nodes**				
Median (Range)	3 (1; 30)	3 (1; 21)	3 (1; 30)	0.802
1-3	284 (58%)	135 (58%)	149 (58%)	0.701
4-9	163 (33%)	80 (34%)	83 (32%)	
≥10	43 (9%)	18 (8%)	25 (10%)	
**Histologic subtype**				
Lobular	77 (16%)	31 (13%)	46 (18%)	0.363
Ductal	362 (74%)	178 (76%)	184 (72%)	
Other	51 (10%)	24 (10%)	27 (10%)	
**Tumor Grade**				
I	68 (15%)	31 (14%)	37 (15%)	0.428
II	235 (51%)	106 (48%)	129 (53%)	
III	159 (34%)	82 (37%)	77 (32%)	
Missing	28	14	14	
**Hormone Receptor Status**				
ER+/PR+	311 (64%)	144 (62%)	167 (65%)	**0.040**
ER+/PR-	156 (32%)	83 (36%)	73 (28%)	
ER-/PR+	23 (5%)	6 (3%)	17 (7%)	
**HER2 Status**				
Negative	443 (91%)	209 (90%)	234 (92%)	0.279
Positive	43 (9%)	24 (10%)	19 (8%)	
Missing	4	0	4	
**SET_ER/PR_ Index**				
Median (Q1; Q3)	1.42 (1.02; 1.77)	1.38 (0.98; 1.73)	1.48 (1.05; 1.81)	0.113
**Recurrence Score**				
Median (Q1; Q3)	21.33 (15.06; 38.09)	22.67 (15.70; 42.32)	20.87 (13.92; 35.21)	**0.029**
Low RS (≤25)	288 (59%)	131 (56%)	157 (61%)	0.274
High RS (>25)	202 (41%)	102 (44%)	100 (39%)	

Abbreviations: ER, estrogen receptor; PR, progesterone receptor; IHC, immunohistochemistry; FEC, 5-fluorouracil, epirubicin, and cyclophosphamide; FEC + D, 5-fluorouracil, epirubicin, and cyclophosphamide followed by docetaxel; Q1, first quartile; Q3, third quartile; RS, Recurrence Score; SET_ER/PR_, endocrine-related transcriptional activity index.

### 3.2 Association of SET_ER/PR_ index with clinical and pathologic characteristics

Median SET_ER/PR_ index did not differ by treatment arm (Table 1). Using SET_ER/PR_ index below 1.50 to define SET_ER/PR_ as low in 272 of 490 subjects (56%), we found no association between low SET_ER/PR_ status and median tumor size (p = 0.119), histologic type defined as ductal, lobular or other (p = 0.976), number of involved lymph nodes (p = 0.399), patient age (p = 0.943), age relative to 50 (p = 0.282), or menopausal status (p = 0.158). Low SET_ER/PR_ status was more common in tumors larger than 20 mm (52% versus 48%, p = 0.043) and histologic grade 3 (41% versus 27%, p = 0.007). As expected, low SET_ER/PR_ status was more common when the cancer was PR-negative (42% versus 20%, p < 0.001), HER2-positive (15% versus 1%, p < 0.001) or had high proliferation rate with Ki67 expression in >20% of cancer cells (30% versus 12%, p < 0.001).

### 3.3 Interaction between treatment and biomarker– continuous genomic indices

**Fig. 2:**
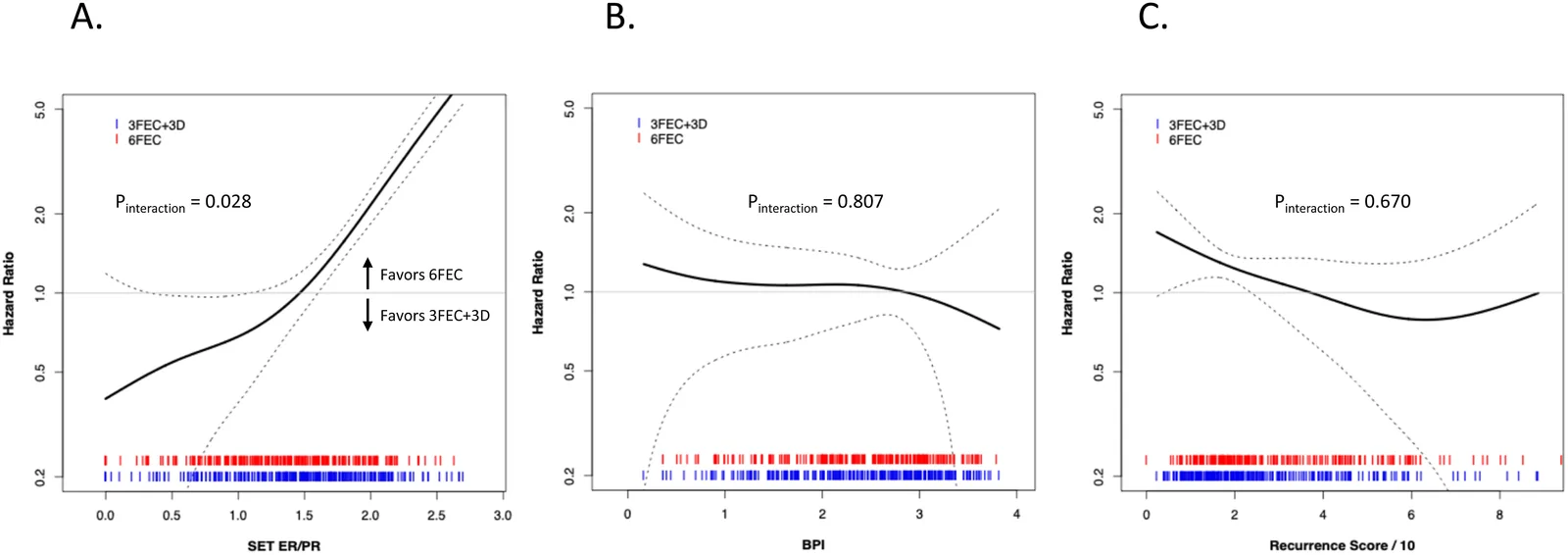
Hazard function plots of the log rate of the hazard for distant recurrence in the 3FEC+3D treatment arm relative to the 6FEC treatment arm as a function of: A) SET_ER/PR_ index; B) baseline prognostic index (BPI); and C) Recurrence Score; with each genomic index presented as a continuous variable, with 95% confidence interval bounds represented by dashed lines. Hazard ratio (values below 1.0 favor 3FEC+3D) have been presented on the y-axis as the linear results but shown on a natural logarithmic (log_n_) scale. The distribution of results from each index has been presented above the x-axis in linear scale to show the values within the 6FEC treatment arm (red) and 3FEC+3D treatment arm (blue).

There was a significant predictive interaction between the SET_ER/PR_ index of endocrine transcriptional activity and chemotherapy treatment arm for DRFI (p_interaction_ = 0.028) that appeared to show relative benefit from 6FEC when SET_ER/PR_ index was higher (Fig. 2A). The relative hazard function curve crossed the line of parity at approximately SET_ER/PR_ index of 1.5 (Fig. 2A). The predictive interaction term was not significant for overall survival (p_interaction_ = 0.224). Neither the baseline prognostic index (p_interaction_ = 0.807 for DRFI and 0.108 for OS) nor the Recurrence Score (p_interaction_ = 0.670 for DRFI and 0.482 for OS) predicted outcomes between the treatment arms (Fig. 2B and C).

### 3.4 Prediction of treatment effect – biomarker status, clinical covariates

**Fig. 3:**
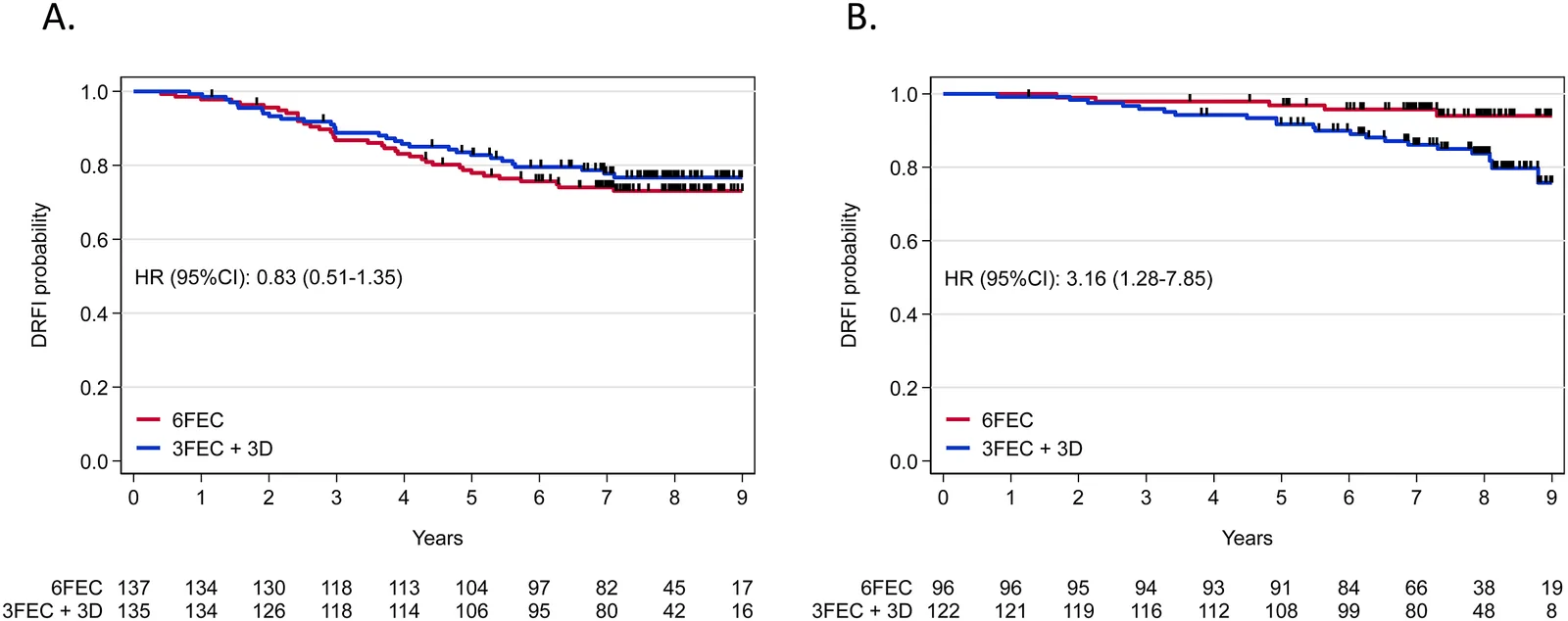
Kaplan-Meier plot of DRFI by chemotherapy treatment arm in the patient subsets defined by: A) low endocrine activity (SET_ER/PR_ < 1.50); B) high endocrine activity (SET_ER/PR_ ≥ 1.50). Abbreviations: SET_ER/PR_, endocrine-related transcriptional activity index; 6FEC, six cycles of 5-fluorouracil, epirubicin, and cyclophosphamide; 3FEC+3D, three cycles of 5-fluorouracil, epirubicin, and cyclophosphamide followed by three cycles of docetaxel; HR: hazard ratio; CI, confidence interval.

**Fig. 4:**
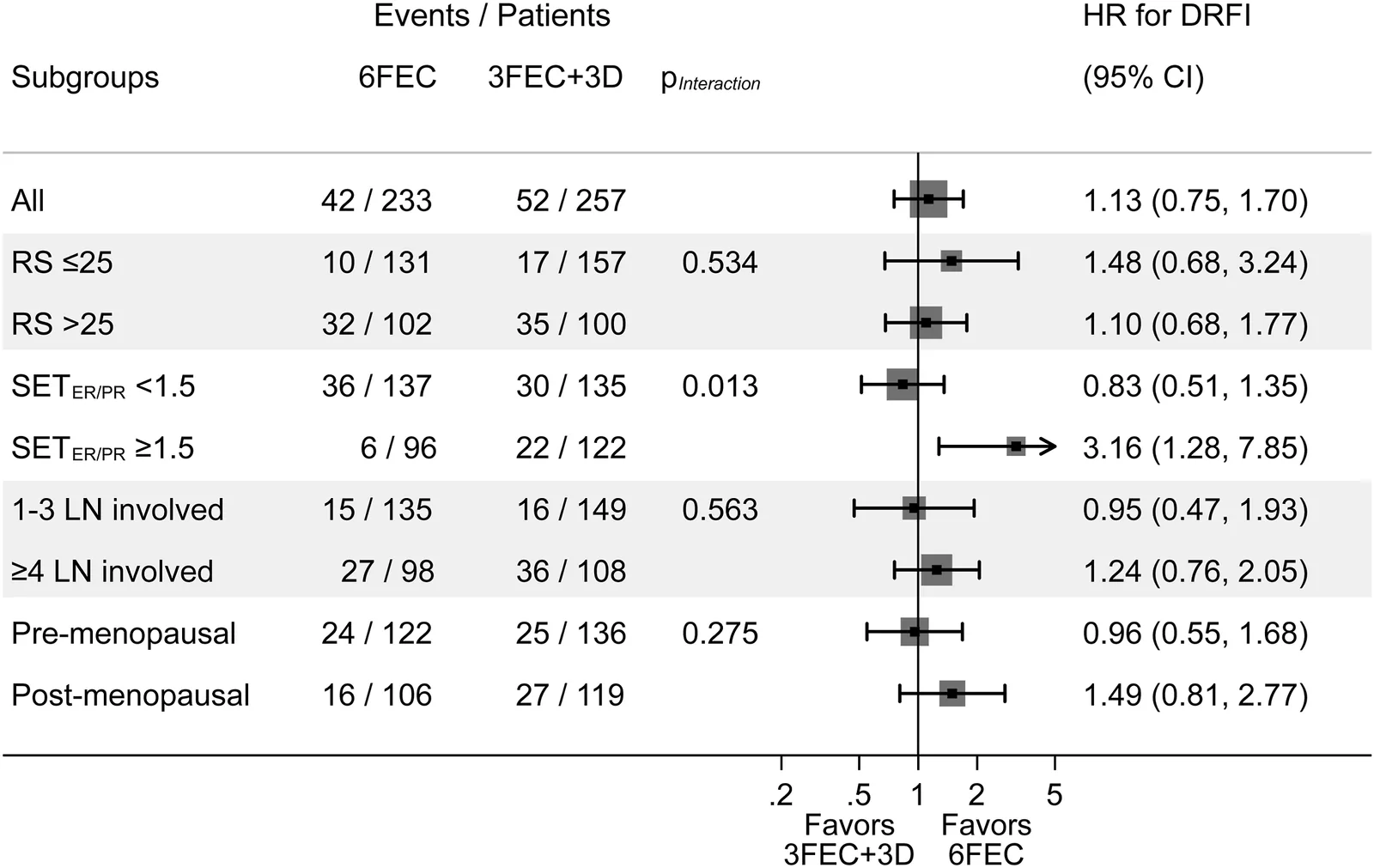
Forest plot of treatment effect on DRFI overall and for each of the following variables: RS status, SET_ER/PR_ index status, lymph nodes (1-3, ≥4), and menopausal status. Abbreviations: SET_ER/PR_, endocrine-related transcriptional activity index; RS, recurrence score; 6FEC, six cycles of 5-fluorouracil, epirubicin, and cyclophosphamide; 3FEC+3D, three cycles of 5-fluorouracil, epirubicin, and cyclophosphamide followed by three cycles of docetaxel; HR: hazard ratio; CI, confidence interval.

There was no statistical interaction between tumoral SET_ER/PR_ index below 0.75 and chemotherapy treatment arm on DRFI (p_interaction_ = 0.668). However, there was significant interaction between SET_ER/PR_ status (<1.50, versus ≥1.50) and chemotherapy treatment arm on DRFI (p_interaction_ = 0.013). There was no significant difference in DRFI observed between treatment arms when SET_ER/PR_ index was below 1.50 (HR 0.83, 95%CI 0.51–1.35), although the curves did not overlap (Fig. 3A). However, the patients with SET_ER/PR_ index 1.50 or higher (Fig. 3B) had significantly longer DRFI after 6FEC compared to 3FEC+3D (HR 3.16, 95%CI 1.28–7.85). Analyses of the other biomarker and clinical covariates demonstrated no predictive interaction between RS status (RS ≤ 25; >25), nodal burden (1–3; ≥4 positive lymph nodes), or menopausal status and the assigned chemotherapy treatment arm on DRFI (Fig. 4). We note that the treatment arms in premenopausal women were similarly balanced according to SET_ER/PR_ status (≥1.50 versus <1.50): 53.3% with high SET_ER/PR_ status and 52.2% with low SET_ER/PR_ status received 3FEC+3D.

### 3.5 Exploratory analysis of SET_ER/PR_ prediction within subgroups

**Fig. 5:**
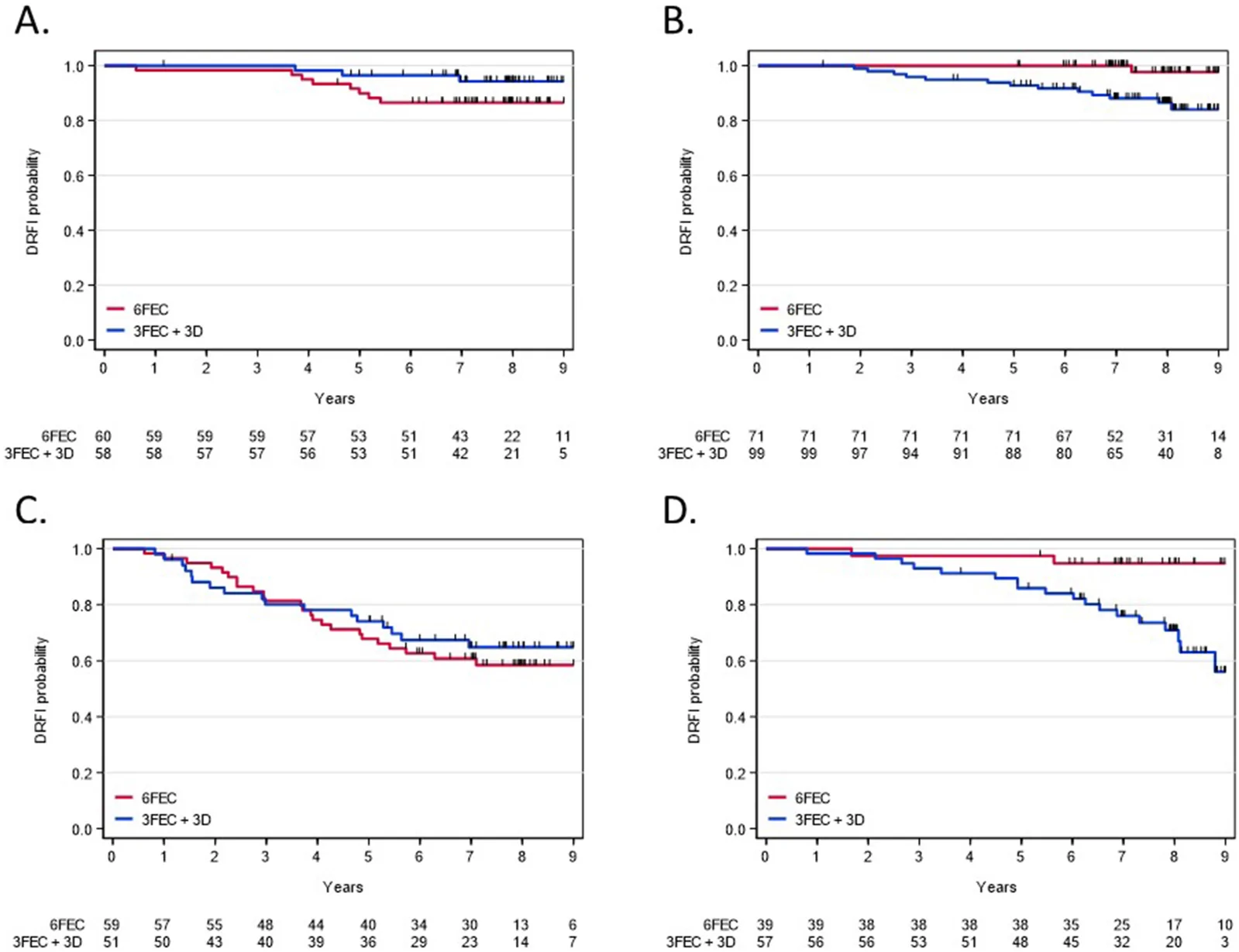
Exploratory analysis of DRFI by chemotherapy treatment arm in subsets of patients defined by: A) low Recurrence Score (RS ≤ 25) and low endocrine activity (SET_ER/PR_ < 1.5); B) low Recurrence Score and high endocrine activity (SET_ER/PR_ ≥ 1.5); C) ≥4 positive lymph nodes and low endocrine activity (SET_ER/PR_ < 1.5); and D) ≥4 positive lymph nodes and high endocrine activity (SET_ER/PR_ ≥ 1.5). Abbreviations: SET_ER/PR_, endocrine-related transcriptional activity index; RS, recurrence score; 6FEC, six cycles of 5-fluorouracil, epirubicin, and cyclophosphamide; 3FEC+3D, three cycles of 5-fluorouracil, epirubicin, and cyclophosphamide followed by three cycles of docetaxel.

There was statistically significant interaction between high SET_ER/PR_ status (≥1.5) and treatment arm on DRFI in two subgroups with low Recurrence Score (p_interaction_ = 0.008) or ≥4 positive lymph nodes (p_interaction_ = 0.009), and their survival plots are presented in Fig. 5. No significant interaction was found in the other subgroups of high RS (>25), 1–3 positive nodes, pre- or post-menopausal status.

## 4 Discussion

Long-term follow up of the PACS-01 trial demonstrated no overall survival benefit by treatment arm in the subset of 1366 patients with HR-positive breast cancer who received adjuvant ET (tamoxifen) [19]. Similarly, we also observed no difference in DRFI between chemotherapy treatment arms within our biomarker cohort of 490 patients with HR-positive breast cancer who received adjuvant ET. However, the SET_ER/PR_ index of endocrine transcriptional activity did predict treatment benefit by adjuvant chemotherapy regimen. Specifically, inclusion of docetaxel (3FEC+3D) was less effective than continued anthracycline-based treatments (6FEC) when the cancer had high endocrine activity (SET_ER/PR_ index at least 1.50).

One interpretation is that breast cancers with high endocrine activity are relatively resistant to docetaxel, compared to anthracycline-based chemotherapy. The cytoskeletal and metabolic consequences of high endocrine activity in breast cancer cells probably render docetaxel less cytotoxic, allowing cellular recovery, as has been described with paclitaxel [15,26,27]. One can also postulate that anthracyclines might elicit a sequential synergy with subsequent ET when the cancer has high endocrine activity. For example, it was reported that overall survival in the 6FEC treatment arm was significantly worse in the subset of patients with HR-positive cancer who did not receive adjuvant ET [19]. This idea is not necessarily specific to anthracycline treatments and it might also apply to other non-taxane treatments, since a breast cancer's response to chemotherapy and its sensitivity to the endocrine therapy that follows have additive prognostic effects [28].

The EBCTCG meta-analysis clearly showed that different chemotherapy regimens have different efficacy for patients with hormone receptor-positive breast cancer [3]. Thus, specific chemo-predictive biomarkers might inform which chemotherapy regimen to offer an individual patient. Recurrence score is the most-validated genomic biomarker for general chemo-prediction, identifying patients in the TAILORx and RxPONDER trials who were unlikely to benefit from chemotherapy based on physician's choice of regimen [1,2]. In other studies, predictive interaction was demonstrated for benefit from CMF chemotherapy in the NSABP B-20 trial and anthracycline-based chemotherapy in the SWOG 8814 trial, versus no chemotherapy [4,5]. On the other hand, SET_ER/PR_ index did not predict benefit from anthracycline-based chemotherapy in the SWOG 8814 trial, but is the only genomic biomarker that has demonstrated prediction of survival outcomes between chemotherapy regimens for hormone receptor-positive cancer, in trials that included taxane chemotherapy [[15], [16], [17]]. Hence, these predictive biomarkers (RS and SET_ER/PR_ index) performed differently in PACS-01, most likely because SET_ER/PR_ index identified patients for whom docetaxel was less efficacious.

Comparing this study with other recent work suggests that a predictive interaction between SET_ER/PR_ index in the tumor and taxane-containing chemotherapy might be different for docetaxel and paclitaxel [15,16]. The cut point of 0.75 was predictive for contemporary dose-intense paclitaxel treatments in the CALGB 96741 and confirmed in the GEICAM/9906 trial, but was not predictive with docetaxel in PACS-01 [15,16]. Rather, a cut point of 1.50 was predictive of outcome related to inclusion of docetaxel in the PACS-01 trial. However, we also note that docetaxel was not administered with a dose-intense schedule due to relative intolerability [1,29]. The predictive relationship between SET_ER/PR_ index deserves further investigation in other trials that compared taxane treatments.

A limitation of this study is that the sample size is small for a test of predictive interaction. Nevertheless, we did observe a significant statistical interaction between treatment arm and SET_ER/PR_ index as a continuous variable and identified a cut point of 1.50. However, the non-significant qualitative separation of survival curves for patients with SET_ER/PR_ index below 1.50 might be the result of an underpowered analysis. Statistical power was further limited for subset analyses, especially because the greater treatment effect was seen in cancers with high SET_ER/PR_ index and/or low RS, which are more endocrine-sensitive and have generally better prognosis with lower event rate. Finally, this study did not exclude HER2+ cancers. SET_ER/PR_ index was below 1.50 in 40 of 43 (95%) known HER2+ cancers in our study. We can't rule out the possibility that inclusion of HER2+ cancers might have confounded the results in the low SET_ER/PR_ index subset if anthracycline treatments are more effective than docetaxel in HR+/HER2+ cancers.

In summary, SET_ER/PR_ index was the first biomarker to predict benefit from taxane-based chemotherapy in HR + breast cancer, and this study from the PACS-01 trial is the first to demonstrate a predictive interaction between treatment and biomarker when the taxane is docetaxel. To date, PACS-01 is the third study to report a predictive interaction between SET_ER/PR_ index and benefit from a taxane-containing chemotherapy [15,16]. Importantly, that predictive interaction appears to be different with docetaxel from that seen with paclitaxel. Docetaxel was less effective when SET_ER/PR_ index was high, with possible (but unproven) advantage from docetaxel when SET_ER/PR_ index was low. This study adds further scientific evidence that specific chemo-predictive biomarkers can inform which chemotherapy to recommend to a patient.

## Data sharing statement

De-identified clinical data elements (treatment arm and follow up information) and corresponding tumoral SET_ER/PR_ index values can be obtained through application to the UNICANCER translational research program and approval as a data analysis project. contact_transla@unicancer.fr.

## Study type

Prospective-retrospective study of a predictive biomarker using samples from a randomized clinical trial.

## Disclosures

FP-L reports personal financial interest from AstraZeneca, Bayer, EISAI, Exact Sciences, Gilead, Illumina, Lilly, Menarini/Stemline, MSD, Myriad, Novartis, Pierre-Fabre, Pfizer, Roche, Veracyte; Institutional: research grants from AstraZeneca, Illumina, Myriad, Nanostring; Support for attending meetings and/or travel from AstraZeneca, Gilead, Lilly, MSD, Novartis, Pierre Fabre, Pfizer, Roche. Given her role as Specialty Editor, Frederique Penault-Llorca had no involvement in the peer-review of this article and has no access to information regarding its peer review.

AL, TF, EC and KT declare no competing interests.

RB has employment at Exact Sciences and has no other competing interests.

FD reports Conference invitation from Gilead, Novartis, Daiichi Sankyo, Astra Zeneca, Pfizer; institutional research funding from Gilead, Novartis, Daiichi Sankyo, Astra Zeneca, Pfizer, Lilly, Roche, Menarini MSD.

ML-T reports consulting, lectures from AstraZeneca, Daiichi Sankyo, MSD, Roche, Roche Diagnostics, Exact Sciences.

TB reports institutional research funding from Astra Zeneca, Pfizer, SeaGen (now Pfizer), Novartis; Payment or honoraria for lectures, presentations, speakers bureaus, manuscript writing or educational events from Astra Zeneca, Daiichi, Novartis, Pfizer, Lilly; Support for attending meetings and/or travel from Roche, Daiichi, Astra Zeneca, Pfizer, Novartis; Participation on a Data. Safety Monitoring Board or Advisory Board for Astra Zeneca, Daiichi, Seagen, Novartis, Pfizer, Lilly.

FA reports personal financial interest from Lilly France, Relay Therapeutics as advisory board member; Institutional relationship with advisory board with AstraZeneca, Daiichi Sankyo, Roche, Lilly, Pfizer, Owkin, Novartis, Guardan Health, N-Power Medecine, Servier, Gilead, Boston Pharmaceuticals, Relay Therapeutics; institutional research funding from Novartis, Pfizer, Roche, Daiici Sankyo, Guardan Health, Owkin.

PB and JL declare no competing interests.

WFS reports patents co-inventoring (US Patent No. 11,459,617, applicant proprietor: University of Texas MD Anderson Cancer Center; Pending Patent No. WO2024227177, applicant proprietor: University of Texas MD Anderson Cancer Center) licensed to Delphi Diagnostics; consulting fees from AstraZeneca; stock and other ownership interests from IONIS Pharmaceuticals and Delphi Diagnostics; and uncompensated scientific advisor relationship with Delphi Diagnostics.

## Role of the funding source

The analysis was supported in part by grant awards from the 10.13039/100004917Cancer Prevention and Research Institute of Texas (RP#180712 to WFS) and 10.13039/100001006Breast Cancer Research Foundation (BCRF-158 to WFS), and by the Ligue Nationale Contre le Cancer, Sanofi-Aventis (France), 10.13039/100004319Pfizer (France), and 10.13039/100002429Amgen (France).

## CRediT authorship contribution statement

**Frédérique Penault-Llorca:** Conceptualization, Data curation, Formal analysis, Investigation, Supervision, Validation, Visualization, Writing – original draft, Writing – review & editing. **Amelie Lusque:** Conceptualization, Data curation, Formal analysis, Investigation, Methodology, Supervision, Validation, Visualization, Writing – original draft, Writing – review & editing. **Thomas Filleron:** Formal analysis, Writing – review & editing. **Kevin Tran:** Formal analysis, Investigation, Methodology, Writing – review & editing. **Lili Du:** Formal analysis, Investigation, Methodology, Writing – review & editing. **Rick Baehner:** Formal analysis, Validation, Writing – review & editing. **Florence Dalenc:** Formal analysis, Validation, Writing – original draft. **Magali Lacroix-Triki:** Formal analysis, Validation, Writing – review & editing. **Thomas Bachelot:** Formal analysis, Supervision, Validation, Writing – review & editing. **Fabrice Andre:** Formal analysis, Validation, Writing – review & editing. **Pascal Boucher:** Formal analysis, Project administration, Validation, Writing – review & editing. **Jérôme Lemonnier:** Formal analysis, Funding acquisition, Project administration, Resources, Validation, Writing – review & editing. **W. Fraser Symmans:** Conceptualization, Data curation, Formal analysis, Funding acquisition, Investigation, Methodology, Supervision, Validation, Visualization, Writing – original draft, Writing – review & editing.

## Declaration of interest statement

Frédérique Penault-Llorca declare the following financial interests/personal relationships which may be considered as potential competing interests:reports personal financial interest from AstraZeneca, Bayer, EISAI, Exact Sciences, Gilead, Illumina, Lilly, Menarini/Stemline, MSD, Myriad, Novartis, Pierre-Fabre, Pfizer, Roche, Veracyte; Institutional: research grants from AstraZeneca, Illumina, Myriad, Nanostring; Support for attending meetings and/or travel from AstraZeneca, Gilead, Lilly, MSD, Novartis, Pierre Fabre, Pfizer, Roche. Given her role as Specialty Editor, Frederique Penault-Llorca had no involvement in the peer-review of this article and has no access to information regarding its peer review.

Amelie Lusque declare that she has no known competing financial interests or personal relationships that could have appeared to influence the work reported in this paper.

Thomas Filleron declare that he has no known competing financial interests or personal relationships that could have appeared to influence the work reported in this paper.

Kevin Tran declare that he has no known competing financial interests or personal relationships that could have appeared to influence the work reported in this paper.

Lili Du declare that she has no known competing financial interests or personal relationships that could have appeared to influence the work reported in this paper.

Rick Baehner declare that he has no known competing financial interests or personal relationships that could have appeared to influence the work reported in this paper.

Florence Dalenc declares the following financial interests/personal relationships which may be considered as potential competing interests:reports Conference invitation from Gilead, Novartis, Daiichi Sankyo, Astra Zeneca, Pfizer; institutional research funding from Gilead, Novartis, Daiichi Sankyo, Astra Zeneca, Pfizer, Lilly, Roche, Menarini MSD.

Magali Lacroix-Triki declares the following financial interests/personal relationships which may be considered as potential competing interests:consulting, lectures from AstraZeneca, Daiichi Sankyo, MSD, Roche, Roche Diagnostics, Exact Sciences.

Thomas Bachelot declares the following financial interests/personal relationships which may be considered as potential competing interests:institutional research funding from Astra Zeneca, Pfizer, SeaGen (now Pfizer), Novartis; Payment or honoraria for lectures, presentations, speakers bureaus, manuscript writing or educational events from Astra Zeneca, Daiichi, Novartis, Pfizer, Lilly; Support for attending meetings and/or travel from Roche, Daiichi, Astra Zeneca, Pfizer, Novartis; Participation on a Data.

Safety Monitoring Board or Advisory Board for Astra Zeneca, Daiichi, Seagen, Novartis, Pfizer, Lilly.

Fabrice André declares the following financial interests/personal relationships which may be considered as potential competing interests:Personal financial interest from Lilly France, Relay Therapeutics as advisory board member; Institutional relationship with advisory board with AstraZeneca, Daiichi Sankyo, Roche, Lilly, Pfizer, Owkin, Novartis, Guardan Health, N-Power Medecine, Servier, Gilead, Boston Pharmaceuticals, Relay Therapeutics; institutional research funding from Novartis, Pfizer, Roche, Daiici Sankyo, Guardan Health, Owkin.

Pascal Boucher declare that he has no known competing financial interests or personal relationships that could have appeared to influence the work reported in this paper.

Jérôme Lemonnier declare he has have no known competing financial interests or personal relationships that could have appeared to influence the work reported in this paper.

Frazer Symmans declares the following financial interests/personal relationships which may be considered as potential competing interests:Royalties or licences from Delphi Diagnostics; consulting fees from AstraZeneca; patents co-inventoring (US Patent No.11,459,617, applicant proprietor: University of Texas MD Anderson Cancer Center; Pending US Patent No.63/499,035, applicant proprietor: University of Texas MD Anderson Cancer Center); stock and other ownership interests from IONIS Pharmaceuticals and Delphi Diagnostics; research funding (institution) from Pfizer; and uncompensated scientific advisor relationship with Delphi Diagnostics.
