# Supplementary material for: Prediction of benefit from docetaxel chemotherapy for HR-positive breast cancer in the PACS-01 trial
Source: Breast. 2026 Aug 7;89:104894. doi: 10.1016/j.breast.2026.104894 (PMC13488032; doi:10.1016/j.breast.2026.104894)
Supplement: Supplementary file 1 [file mmc1.pdf]

Statistical Analysis Plan (SAP)

Evaluation of Predicted Sensitivity to Endocrine Therapy (SET) in Primary Tumor Samples from the PACS-01 Trial

Version n°1.4 – July 11, 2024

Coordinating Investigator:

- William Fraser Symmans, University of Texas MD Anderson Cancer Center, email: fsymmans@mdanderson.org
- Frédérique Penault-Llorca, Centre Jean PERRIN, Clermont-Ferrand, email: Frederique.PENAULT-LLORCA@clermont.unicancer.fr

Methodologist: Thomas Filleron, Oncopole Claudius Regaud, IUCT-O, Toulouse

Biostatistician: Amélie Lusque, Oncopole Claudius Regaud, IUCT-O, Toulouse

APPROVAL AND SIGNATORIES OF THE STATISTICAL ANALYSIS PLAN

	Name	Date (dd/mm/yyyy)	Signature
Coordinating Investigator	William Fraser Symmans	21/07/2026	William Fraser Symmans ✓ Certified by youtrust
Coordinating Investigator	Frédérique Penault-Llorca	20/07/2026	Frédérique PENAULT-LLORCA ✓ Certifié par youtrust
Unicancer	Jérôme Lemonnier	27/07/2026	Jerôme Lemonnier ✓ Certifié par youtrust
Methdologist	Thomas Filleron	20/07/2026	Thomas FILLERON ✓ Certifié par youtrust

TABLE OF CONTENTS

1. INTRODUCTION 4
 1.1 PACS-01 TRIAL 4
 1.2 SAMPLE SIZE FOR THIS PROJECT 4
 1.3 RECURRENCE SCORE 4
 1.4 SET BIOMARKER 5
2. OBJECTIVES 6
 2.1 PRIMARY OBJECTIVE 6
 2.2 SECONDARY OBJECTIVES 6
3. ENDPOINTS 7
 3.1 PRIMARY ENDPOINT 7
 3.2 SECONDARY ENDPOINTS 7
4. STATISTICAL CONSIDERATIONS 8
 4.1 POPULATIONS TO BE ANALYZED 8
 4.2 PATIENT DISPOSITION 8
 4.3 BASELINE DATA 8
 4.4 PRIMARY OBJECTIVE 8
 4.5 SECONDARY OBJECTIVES 9
  4.5.1 *Prognostic evaluation of RS (continuous) and the SET2,3 index (continuous) on DRFI* 9
  4.5.2 *$SET_{ER/PR}$ index predicts benefit from FEC-D compared to FEC* 9
  4.5.3 *Prognosis value of the individual components (RS, $SET_{ER/PR}$ index, and BPI)* 10
  4.5.4 *Relationship between Biomarkers and Clinical and Pathology Variables with DRFI* 10
 4.6 HANDLING OF MISSING DATA 10

1. INTRODUCTION

This document describes the statistical analyses planned for the project “Evaluation of Predicted Sensitivity to Endocrine Therapy (SET) in Primary Tumor Samples from the PACS-01 Trial”. The statistical analyses will be carried out using STATA 18 software at the Oncopole Claudius Regaud IUCT-Oncopole (Biostatistics & Health Data Science Unit).

This document contents a brief description of the PACS-01 trial, biomarkers and the statistical methodology that will be used for this project.

1.1 PACS-01 TRIAL

The PACS 01 trial was a phase III clinical triaal that compared six cycles of fluorouracil, epirubicin, and cyclophosphamide (FEC) with a sequential regimen of three cycles of FEC followed by three cycles of docetaxel (FEC-D) as adjuvant treatment for women with node-positive early breast cancer. Some patients with hormone receptor-positive (HR+) primary breast cancer from the PACS-01 trial did not receive adjuvant endocrine therapy, according to treatment standards from that time.

1.2 SAMPLE SIZE FOR THIS PROJECT

This project will be performed in 792 patients with hormone receptor-positive (HR+) primary breast cancer from the PACS-01 trial.

1.3 RECURRENCE SCORE

Recurrence Score (OncotypeDX) (RS) was demonstrated in the SWOG S-8814 trial to predict benefit from anthracycline-based chemotherapy (4 cycles of CAF) in addition to tamoxifen endocrine therapy for node-positive breast cancer. A recent evaluation of RS in samples from PACS-01 demonstrated that RS was prognostic after chemo-endocrine adjuvant therapy in both treatment arms, but did not predict benefit from docetaxel when the randomized treatment arms were compared (i.e no predictive interaction between RS and FEC-D versus FEC). Contemporary opinion holds that the cutpoint of RS>25 has identifies “chemo-sensitive” disease, although that has not been well validated with current chemotherapy regimens. Recently, the RxPONDER trial reported that the cutpoint of RS≤25 identified lack of chemotherapy benefit for post-menopausal women, whereas there was chemotherapy benefit for pre-menopausal women.

1.4 SET BIOMARKER

SET2,3 was designed to measure endocrine-related transcriptional activity in a tumor and adjust the measurement for the baseline prognostic risk due to disease burden and subtype. Therefore, a sensitivity analysis of this biomarker will be performed in the population who received adjuvant endocrine therapy following adjuvant chemotherapy. SET2,3 is calculated from the weighted sum of $SET_{ER/PR}$ index of hormone receptor-related transcription (excluding proliferation genes) and a baseline prognostic index (BPI) including tumor and nodal stage (pathologic tumor diameter and number of involved lymph nodes) and molecular subtype (RNA4) from gene expression measurements of ER, PR, HER2 and aurora kinase A. The concept behind development of the $SET_{ER/PR}$ index was to identify a signature of gene transcription that reliably represents the genomic activity of ER and PR activity in breast cancer, but to exclude genes that are obviously related to cell cycle proliferation, hence avoiding the usual paradoxical association between prognostic classifiers and patient outcomes that attributable to genes associated with proliferation.

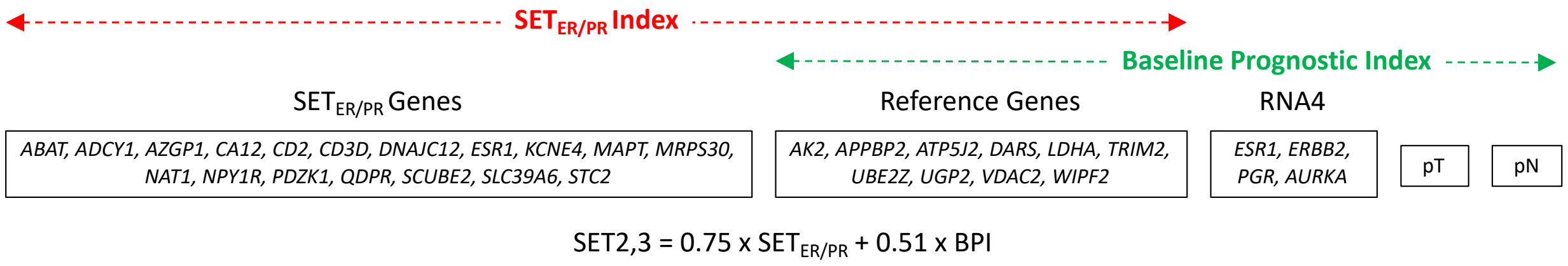

Figure 1. The SET2,3 test measures the $SET_{ER/PR}$ Index of non-proliferative transcription related to both ER and PR, then adjusts for the baseline prognostic risk that is derived from the pathologic tumor size (pT), number of involved lymph nodes (pN), and the RNA4 prognostic classifier.

SET2,3 is accurately measured from FFPE tissue sections using the selected diagnostic platform (QuantiGene Plex (QGP) assay, Thermo Fisher). Additionally, these measurements are highly reproducible within and between laboratories.

Clinical validation studies have demonstrated that $SET_{ER/PR}$ index is independently prognostic when endocrine therapy is used to treat relapsed metastatic disease, or when adjuvant endocrine therapy is administered after neoadjuvant chemotherapy where it adds information to residual cancer burden after the chemotherapy. SET2,3 index (and the $SET_{ER/PR}$ component) predicted early pharmacodynamic response to neoadjuvant endocrine therapy and long-term prognosis in ACOSOG Z-1031 (Symmans et al ASCO 2021).

In the SWOG S8814 trial, OncotypeDx Recurrence Score (performed at Genomic Health Inc.) was not correlated with SET2,3: approximately half had high SET2,3 (≥2.10) when recurrence score was ≤25 or >25. SET2,3 index, and each component index ($SET_{ER/PR}$ index and BPI), added independent prognostic information to recurrence score during the first 5 years (during adjuvant endocrine therapy) but only the BPI was prognostic after 5 years. SET2,3 and recurrence score were complementary biomarkers in S8814, including patients whose adjuvant treatment was tamoxifen for 5 years alone. The S-8814 result is interesting, given that RS is known to have endocrine predictive capability. Our interpretation is that SET2,3 was complementary due to added endocrine predictive performance due to the $SET_{ER/PR}$ index in addition to the inclusion of tumor size and nodal information.

2. OBJECTIVES

2.1 PRIMARY OBJECTIVE

The primary objective of this project is to confirm whether the combination of SET2,3 index (using cutpoint of 2.10) and Recurrence Score (using cutpoint of 25) adds prognostic information to Recurrence Score alone in the PACS-01 trial.

2.2 SECONDARY OBJECTIVES

The secondary objectives of this project are:

- To compare the prognostic performance of SET2,3 (continuous or using cutpoint of 2.10) and Recurrence Score (continuous or using cutpoint of 25) in PACS-01.
- To determine whether $SET_{ER/PR}$ index predicts benefit from FEC-D compared to FEC in the PACS-01 trial.
- To identify an optimal range of $SET_{ER/PR}$ index values to predicted benefit from FEC-D (versus FEC) in the PACS-01 trial.
- To compare prognostic and predictive associations in pre- and post-menopausal women.

3. ENDPOINTS

3.1 PRIMARY ENDPOINT

The primary endpoint is distant recurrence free interval (DRFI), defined as the time from randomization to distant recurrence. Patient without event will be censored at the end of follow-up or death if no distant recurrences occur.

3.2 SECONDARY ENDPOINTS

Disease-free survival (DFS) is defined as the time from randomization to first local, regional or distant recurrence, contralateral breast cancer, or death from any cause. Patient without event will be censored at the end of follow-up.

Overall Survival (OS) is defined as the time from randomization until death from any cause. Patients who are alive at last follow-up news will be censored at this date.

Study Endpoint Definitions

Study Endpoint	Local Regional Recurrence	Distant Recurrence	Contralateral Breast Cancer	Death From Any Cause
Distant Recurrence Free Interval		Event		Censor
Disease Free Survival	Event	Event	Event	Event
Overall Survival				Event

4. STATISTICAL CONSIDERATIONS

4.1 POPULATIONS TO BE ANALYZED

- Main Analysis Population: All samples that are evaluable for each objective.
- Sensitivity Population: Subjects who received adjuvant endocrine therapy.

4.2 PATIENT DISPOSITION

Patient disposition will be summarized on the overall population as follows:

- Number of patients in the PACS-01
- Patients clinically eligible
- Patients with tissue block
- Fixative: Bouin vs Non Bouin tumor tissue fixation
- RNA sample provided to MDACC laboratory
- SET assay result met all Quality Control (QC) requirements
- Analysis of Main Population: subjects with both SET2,3 and RS results for primary objective; subjects with $SET_{ER/PR}$ index result for secondary objective
    - Menopausal status (pre-, post-)
- Analysis of Sensitivity Population: subjects who received adjuvant endocrine therapy
    - Menopausal status (pre-, post-)

A flow-chart describing the patient disposition will be presented in the statistical analysis report for each objective. Patient disposition will be also described according to tumor tissue fixation (Bouin vs Non Bouin).

4.3 BASELINE DATA

Demographic and clinical characteristics will be presented in the main analysis and sensitivity population using usual statistics. Quantitative data will be summarized as median, min, max and number of missing data. Qualitative variables will be described as number, percentage and number of missing data.

4.4 PRIMARY OBJECTIVE

The primary endpoint for this analysis is Distant Recurrence Free Interval (DRFI). Analyses for the primary objective will be performed in all evaluable subjects and repeated in the sensitivity population. For the prognostic evaluation of RS risk group and the SET2,3 index group, all treatment arms will be combined (FEC and FEC-D). First, the prognosis of RS (≤25, >25) and SET2,3 index group (≥2.10, <2.10) will be evaluated individually. The primary objective of this project is to confirm whether the SET2,3 index ≥2.10, <2.10) and Recurrence Score (≤25, >25)] adds prognostic information compared to Recurrence Score alone on DRFI. The association between the SET2,3 index group, RS risk group and DRFI for the pooled treatment groups will be estimated using a Cox proportional hazard regression model. The model will include the main effects for treatment, the Recurrence Score Risk Group (≤25, >25) and the SET2,3 index group (≥2.10, <2.10). The estimated hazard ratio for SET2,3 index group and Recurrence Score will be tested for significance using a Wald test, and will be presented with the corresponding 95% confidence interval.
Likelihood-ratio test will be used to compare the models (adjusted for treatment and RS risk group) with versus without SET2,3 risk group.

Furthermore, underlying assumptions for the Cox proportional hazards model will be evaluated. If these assumptions are violated, then alternative models will be considered as exploratory analyses.

If the SET2,3 index group and RS risk group are both independently prognostic, a multivariable model will be built to evaluate whether there was an additive or synergistic effect. The model will include the main effects for treatment, the Recurrence Score Risk Group (≤25, >25), the SET2,3 index group (≥2.10, <2.10) and interaction term between Recurrence Score Risk Group (≤25, >25) and the SET2,3 index group (≥2.10, <2.10).

4.5 SECONDARY OBJECTIVES

The primary endpoint for this analysis is Distant Recurrence Free Interval (DRFI). Analyses for each secondary objective will be performed in all evaluable subjects and repeated in the sensitivity population.

4.5.1 Prognostic evaluation of RS (continuous) and the SET2,3 index (continuous) on DRFI

For the prognostic evaluation of RS (continuous) and the SET2,3 index (continuous), all treatment arms will be combined (FEC and FEC-D). The association between the SET2,3, RS and DRFI for the pooled treatment groups will be estimated using a Cox proportional hazard regression model. The model will include the main effects for treatment, the Recurrence Score (per 10) and the SET2,3 index (Continuous). The estimated hazard ratio for SET2,3 index and Recurrence Score will be tested for significance using a Wald test, and will be presented with the corresponding 95% confidence interval.
Likelihood-ratio test will be used to compare the models (adjusted for treatment and RS) with versus without SET2,3. Furthermore, underlying assumptions for the Cox proportional hazards model will be evaluated. If these assumptions are violated, then alternative models will be considered as exploratory analyses. Using restricted cubic splines, the linearity of continuous SET2,3 index will be assessed

Two similar analyses will be performed with:
- RS continuous and SET2,3 categorical (≥2.10, <2.10),
- SET2,3 continuous and RS categorical (≤25, >25).

4.5.2 $SET_{ER/PR}$ index predicts benefit from FEC-D compared to FEC

The association between the $SET_{ER/PR}$ (continuous) and DRFI for each treatment group will be estimated using a Cox proportional hazards regression model with main effects for treatment, the $SET_{ER/PR}$ index (continuous), interaction term between the treatment arm and the $SET_{ER/PR}$ index (continuous). Wald tests will be used for assessing significance of the association between $SET_{ER/PR}$ and DRFI within treatment groups, and the estimated hazard ratios will be presented with corresponding 95% confidence intervals. Statistical significance of the interaction between $SET_{ER/PR}$ and treatment group will be assessed using a Wald test for equality of the two log hazard ratios. A p-value for interaction that is below 0.1 will be considered significant, since this is an exploratory analysis in a population with modest sample size.
An additional analysis will model hazard ratio for treatment effect (FEC-D vs FEC) according to $SET_{ER/PR}$ index as hazard function plot with 95% confidence bounds also plotted and two splines to represent nonlinear hazard functions. If an optimal range of $SET_{ER/PR}$ index values to predict benefit from FEC-D versus FEC chemotherapy can be identified from the hazard function plot, then the selected cut point(s) will be applied and Kaplan Meier plots will be used to illustrate the survival curves according to treatment arm within each subset.
Similar analysis will be conducted with $SET_{ER/PR}$ dichotomized: 1) at median (below vs at or above), 2) as within vs outside interquartile range and 3) below 0.75 vs at or above 0.75.

4.5.3 Prognosis value of the individual components (RS, $SET_{ER/PR}$ index, and BPI)

The association between individual components (RS, $SET_{ER/PR}$ index, and BPI) and DRFI will be evaluated using a multivariable Cox proportional hazard Model. The model will include the main effects for treatment, the RS (≤25, >25), the $SET_{ER/PR}$ and BPI. For each term, the estimated hazard ratio will be tested for significance using a Wald test and will be presented with the corresponding 95% confidence interval.

4.5.4 Relationship between Biomarkers and Clinical and Pathology Variables with DRFI

Prognostic and/or predictive associations of RS (≤25, >25) and/or SET2,3 index group (≥2.10, <2.10) will be evaluated according to subjects' menopausal status (pre-, post-) as individual biomarkers and in combination. The association between the SET2,3, RS, menopausal status and DRFI will be estimated using a Cox proportional hazard regression model. Kaplan-Meier survival plots will be prepared.

Nodal burden (1-3, versus ≥4 positive lymph nodes) is another important covariate or subset that is clinically relevant because there are currently no guidelines for use of biomarkers for patients with ≥4 positive nodes.

The association between RS (≤25, >25), SET2,3 index group (≥2.10, <2.10), individual clinical and pathology variables, and DRFI will be examined, including menopausal status (pre, post), age (≤50, >50 years old), number of positive nodes, lymph node status (1-3,>3), study tumor size (mm), study tumor size (≤20mm, >20mm), study tumor grade (I,II, and III), , surgery type (lumpectomy, mastectomy), treatment arm (FEC vs. FEC-D), adjuvant hormonal therapy (yes, no), radiation therapy (yes, no), ER status by IHC (negative, positive), PR status by IHC (negative, positive), Ki-67 status by IHC (≤20%, >20%), and HER2 status by IHC and FISH (negative, positive). A multivariable model will be built using backward variable selection, to identify the variables with independently significant associations with DRFI, using a criterion of p ≤ 0.05.

Similar analysis will be conducted in the main analysis population for Disease Free Survival and Overall Survival. This analysis will be repeated in the sensitivity population.

4.6 HANDLING OF MISSING DATA

The primary method for handling missing time-to-event data will be censoring.

The following conventions will be applied to impute partial date:

- If the day is missing, the 15th or the first or the last day of the month will be used to replace the missing day (depending on the type of the date).
- If the day and month are missing, June 15 or January 1 or December 31 will be used to replace the missing day and month (depending on the type of the date).
- If the day, month, and year are all missing, the date will not be imputed.

Other missing data will not be imputed.
